# Socio-economic determinants of intimate-partner physical violence among women in South Africa

**DOI:** 10.3389/fpsyg.2025.1499263

**Published:** 2025-03-26

**Authors:** Keatlegile Mabena, Wandile Tsabedze, Xolisa Mazibuko

**Affiliations:** Department of Development Studies, University of South Africa, Pretoria, South Africa

**Keywords:** intimate-partner physical violence, South Africa, women, socio-economic, determinants

## Abstract

**Introduction:**

Intimate partner violence (IPV) against women is a worldwide public health and human rights problem that occurs in various forms which includes physical, emotional, and sexual abuse. It is commonly perpetrated by the male intimate partner. This phenomenon cuts across the global South regions, including South Africa. In South Africa, women bear the brunt of intimate-partner physical violence, there is a need to reduce femicide and highlight the preponderance of intimate-partner physical violence in South Africa.

**Methods:**

The main objective is to explore the socio-economic determinants of IPV among women in South Africa. Data are drawn from the 2016 South Africa Demographic and Health Survey 2016. The total sample (8,514) of women aged 15–49 years was selected and interviewed for domestic violence. The sampling method used for the survey conducted was a stratified sample selected in two stages, with enumeration areas (EA) as the sampling units for the first stage. Univariate analysis was performed to show the distribution of the variables in the study followed by bivariate analysis (Pearson's chi-square statistics) showing the relationship between individual variables and the dependent variable.

**Results:**

Using multivariate analysis (Binary regression model) to determine the socio-economic variables revealed the significance of *p* < 0.01–*p* < 0.04, *p* < 0.005–*p* < 0.009 and *p* < 0.005, associated with the dependent variable while controlling for the effects of other variables. The results revealed significant Odds Ratios (ORs) that highest educational attainment, ORs (1.565), wealth index, ORs (poorer = 0.883, middle = 0.924), employment status ORs (1.073), current marital status, ORs (married = 0.425, living together = 0.479, divorced/separated = 0.422), and justifying wife-beating ORs (yes = 3.030).

**Conclusion:**

This shows the need for policymakers to address physical violence by placing an emphasis on formulation of programmes and policies that empower women through education, employment, and political participation.

## Introduction

Physical violence (IPPV) remains to be a global public health crisis that violates basic human rights, causes harm to physical, sexual or psychological wellbeing and has an impact on society. Alkan et al. (2022) and Alkan and Tekmanli (2021) view IPPV as a sociological issue which negatively affects society. Intimate partner violence (IPV) is described as any behavior within a current, extended, or would-be intimate relationship that causes physical, psychological, or sexual harm to either partner (Wessells and Kostelny, 2022; Centers for Disease Control Prevention, 2022; Ogundipe et al., 2018; Lutgendorf, 2019; Warmling et al., 2021; Alkan et al., 2023). Intimate partner violence (IPV) manifests in various forms that include physical, sexual, or emotional (Sardinha et al., 2022; Yang, 2022; Metheny, 2019; Eggers del Campo and Steinert, 2022). These forms of abuse are often influenced by societal and cultural norms, perceived gender roles, and general acceptance of violence within the intimate relationships (Moreira and Da Costa, 2020; Rajah and Osborn, 2022; Di Napoli et al., 2019; Ogbe et al., 2020). A 2009 Medical Research Council study reported that one in five women has experienced physical violence in South Africa. This figure is reported to be higher in the poorest households, where at least one in three women has reported physical violence. Three women die at the hands of their intimate partner every day. The recorded murder rate of 24.7 per 100,000 women in South Africa is significantly higher than global levels. This leads to an increase in femicides rates (South African Medical Research Council, 2019).

Both men and women can be victims of physical violence, although it is the highest among women. Acts of IPPV include: slapping (with open or closed hand), shoving, pushing, punching, hitting, beating, scratching, hair pulling, strangling, biting, spitting, grabbing, shaking, spitting, kicking, burning, throwing, twisting of a body part, forcing the ingestion of an unwanted substance; restraining a woman from seeking medical treatment or other help; and using household objects to hit or stab a woman, using weapons like knives or guns (Warmling et al., 2021; Metheny, 2019; Hall and Hearn, 2019; Bonamigo et al., 2022; Baskan and Alkan, 2023). Women are commonly the brunt of acts of physical violence perpetrated by their male intimate partners (Di Napoli et al., 2019; Ogbe et al., 2020; Mojahed et al., 2022; Smyth et al., 2021). Intimate-partner physical violence IPPV occurs in private (for example, home, bedroom) and public settings (parks or entertainment areas; World Health Organization, 2012; Craig, 2020). Many victims choose not to report incidents of physical abuse because they perceive it to be a private matter (Smyth et al., 2021; Gurm et al., 2020; Perrin et al., 2019). For instance, in a home environment, culture, traditions, and religion contributes to the secrecy of such behavior. Choosing not to report IPPV encourages the perpetrator to continuously be abusive (Mokwena et al., 2023; Johnson et al., 2019). In 1998 South Africa adopted the Domestic Violence Act (Act 116 of 1998) promulgated in 1999, which states that children, women and vulnerable groups must be protected by law from any form of violence. Women victims do experience IPPV due to different factors such as, psychosocial, sexual, socioeconomic, to mention few (De Wet-Billings and Billings, 2024; Mokwena et al., 2023; Hunt et al., 2024).

Physical violence IPPV is a phenomenon that has received more attention around the globe. However, limited research exists that aims to assess socioeconomic determinants [age in 5-year groups, type of place of residence (Mokwena et al., 2023), highest educational attainment (Chemhaka et al., 2023; Maher and Hayes, 2023), wealth index (Liu and Olamijuwon, 2024), employment status (Zharima et al., 2024), provinces/regions, current marital status, ethnicity] that influence IPPV among women aged 15–49 years in South Africa (De Wet-Billings and Billings, 2024). This indicates that Sub-Saharan Africa women experience IPPV perpetuated by diverse determinants. Therefore, this study will focus on the prevalence and nature of IPPV and factors associated with only this form of violence. Considering the complexity of IPPV that is embedded in all parties of society and affects different aspects of life, there is a need for appropriate interventions that should be put in place to prevent and address socio-economic factors such as age, place of residence, highest educational attainment, wealth index, employment status, marital status, race, etc., which influence the prevalence of physical violence in intimate relationships.

South Africa is one of IPPV violence low- and middle-income countries affected by IPPV violence in the sub-Saharan region with a high prevalence rate of IPV. One in five (20.5%) ever partners reported to have ever experienced physical violence by a partner while 6.2% experienced sexual violence (Statistics South Africa, 2022). Despite efforts from government and NGOs to combat IPV, cases of physical abuse continue to increase. The intimate partner murder rate in South Africa was established to be five times higher than the global average according to the World Health Organization (2021). South Africa is one such country in southern Africa where IPV results in homicide (Mbele, 2022). In South Africa, one in five (21%) ever partner women aged 18 and older have experienced physical violence by a partner by any partner [HEAIDS (The Higher Education and Training HIV/AIDS Programme), 2019]. A review of the Demographic Health Survey by United States for International Development (2010)^1^ confirmed that more than 36% of women in sub-Saharan African countries such as South Africa, Malawi, Zimbabwe, Zambia, Uganda have been victims of IPV. Physical partner violence poses a greater risk to women than any other form of violence. For example, studies have shown that globally 38% of women and girls were killed in 2021 and these violent deaths were committed by intimate partners (Kafka et al., 2021; World Health Organization, 2021). According to Wado et al. (2021), teenage girls (15–19 years) and young women (ages 20–24 years) in Southern Africa are part of the population that is vulnerable to IPV as compared to other age cohorts around the world, and this could be attributed to early entry into marriage unions in the region.

Social learning theory proposes that violence is a learnt behavior, and it can be influenced by various socioeconomic factors. According to the theory, individuals who experience coercive parenting or witness parental violence are more inclined to accept the use of violence in their own intimate relationships (Copp et al., 2019; Enaifoghe and Idowu, 2021; Woollett and Thomson, 2016). This theoretical perspective was employed in the study, gave a clear understanding, or answered the question; ever experienced intimate-partner physical violence? This will indicate if women perceive IPPV as a phenomenon driven by gender norms and societal values. This has an influence on how women react or behave toward IPPV in an African context. The objective of the study was to determine the association of socio-economic determinants; age, place of residence (rural and urban), education, wealth index, employment status, regions, marital status, racial group, and perceptions with physical violence of the intimate partner among South African women.

The study is motivated by the need to contribute to reducing femicides by highlighting the extent of physical violence in South Africa. The study contributes to the body of knowledge in social science fields particularly in the South African context. Furthermore, the findings also add to the achievement of the National Development Plan-2030 (NDP) objective, which aims to improve awareness and educational programmes on gender equality, autonomy, and gender-based violence. Although NDP does not specifically focus on intimate partner violence, this study could serve as a baseline showing the necessity of incorporating IPV into its scope in order to promote social protection and the building of safer communities. The results of this study also add to the progress toward achieving Goals 5 and 16 of the Sustainable Development Goals through the condemnation of all forms of violence against women and girls in all private and public spheres, as it interrogates the factors that cause violence. This assists various stakeholder groups (e.g., government, civil society, and private sector) and sectors (e.g., health, environment, and economy) to adopt and deploy multisectoral approaches in addressing intimate partner violence.

## Methods and materials

### Data source

Data analyzed in this study was extracted from the 2016 South Africa Demographic and Health Survey. The primary objective of the SADHS 2016 is to provide up-to-date estimates of basic demographic and health indicators, but also collects data on intimate-partner physical violence. This dataset was conducted by Statistics South Africa (Stats SA) in partnership with the South African Medical Research Council (SAMRC) conducted on behalf of the National Department of Health (NDoH). The SADHS 2016 followed a stratified two-stage sample design with a probability proportional to the size sampling of primary sampling units (PSUs) in the first stage and systematic sampling of dwelling units (DUs) at the second stage. The study focused on women aged 15 to 49 years, sample size (8,514; Chauvet and Vallée, 2020). The population was divided into manageable groups to be able to select the required sample size (8,514). Once the sample was selected, then the population of each cluster was determined.

### Description of the scale or measurement used in the study

In the study we measured various variables which were dichotomous, nominal, ordinal, interval, or ratio. The dichotomous variable were; ever experienced intimate-partner physical violence? male or female, yes or no, residential and whether or not, these variables helped when analyzing proportions within a population. In addition, this is essential for constructing confidence intervals for population proportions. We were coded “1” if the participant agreed with any of the statements and “0” if she did not agree with any of the statements. There were also nominal variables which were; age groups, provinces, and race. Ordinal variable is a categorical variable with ordered values; these variables were; highest educational attainment, wealth index, employment status and current marital status. Finally, interval variables or ratio variables have a significant difference between values, while ratio variables have a clear definition of zero. Interval variables and ratio variables were quantified, which means that they were measured numerically.

### Variables

#### Dependent variable

The dependent variable (physical violence) used in this study constituted four (4) variables, which were measured: whether or not the respondent has ever been pushed, shaken or had something thrown by husband/partner, has ever been kicked or dragged by husband/partner, has ever been strangled or burnt by husband/partner, has ever been threatened by knife/gun or another weapon by husband/partner. The four above variables were combined to form one variable labeled intimate-partner physical violence. This dummy variable was coded “1” if the participant agreed with any of the statements and “0” if she did not agree with any of the statements.

#### Independent variable

The independent variables in the study included age in categories 15–19, 20–24, 30–34, 35–39, 40–44, and 45–49. The place of residence was categorized as “rural” and “urban.” The education variable was arranged as: no education, primary secondary and tertiary education. The wealth index was categorized as: “poorest,” followed by “poorer,” “middle,” “richer,” and the last category was “richest.” The employment status was categorized as “Unemployed” and “Employment.” The regions include the Western Cape, Eastern Cape, Northern Cape, Free State, KwaZulu-Natal, North-West, Gauteng, Mpumalanga, and Limpopo. The marital status was classified as Never married, married, living together, widowed, divorced/separated, and not living together. The racial group was classified as “Black/African participants,” “white participants,” “Colored participants,” and “Indian/Asian participants.” Perception of intimate partner violence (wife beating justified if wife argues with husband) was classified as “Yes” and “No.”

### Data analysis

Data were analyzed at the univariate, bivariate, and multivariate levels using Statistical Package for Social Sciences (SPSS). At the univariate level, the frequency distribution by various socioeconomic variables was profiled. Univariate analysis was used to describe data, summarize the data and finds patterns in the data. At the bivariate level, the Chi-square was used to examine the association between the dependent variable (intimate partner violence) and each independent variable according to their socio-economic and demographic background. The significance level is reduced to 0.05. At the multivariate level, binary regression was used to control for the effects on other variables in the study. This method of analysis was chosen because the dependent variable is dichotomously coded “1” if the participant agreed with any of the statements and “0” if otherwise.

## Results

Table 1 illustrates the percentage distribution of study respondents by different socio-economic variables. The table indicates that 2.9% of the study participants had experienced physical violence. The data in the table equally show that the sample is constituted by a majority of youth participants specifically in age group 15–19 which represents 17.1%. Most of the respondents reside in urban areas (56.4%) compared to those living in urban areas (43.6%). A higher number of respondents (77.3%) attained secondary education. The category with the lowest percentage of participants is made up of those with no education at all (2.2%). With regard to the wealth index, the majority (23%) of the respondents fall under the middle category. The richest category has the lowest (14.1%) proportion of participants. The respondents were classified as employed or unemployed, and the results reveal that most of participants were unemployed, accounting for 67.8%. The employed category consisted of least respondents (32.2%). Most of the respondents (16%) were from KwaZulu-Natal province, followed by Limpopo (13%), Mpumalanga (12.4%), Eastern Cape (12.2%), Gauteng (10.1%), North-West (10.1%), Free State (10%), and Northern Cape (8.4%). Only a few participants are from the Western Cape Province, which accounts for 7.7%. The distribution by marital status shows that the majority of participants were never married with 60.3%, followed by married (21.4%), living together (11.9%), not living together (2.8%), and widowed (2.4%). The category that comprised the least number of participants was divorced/separated with 1.1%. The results also show that the sample constituted in this study is made up of a majority of Black/African participants (86.5%), with Indian/Asian participants accounting for 1%. Most of the participants indicated that it is unacceptable for a husband to beat his wife, representing 96.3%.

**Table 1:** Distribution of study respondents by different socio-economic variables.

**Variable**	**Percentage**	**Total**
**Ever experienced intimate-partner physical violence?**		
No	87.1	3,451
Yes	12.9	510
**Total**	100	3,961
**Age groups**		
15–19	17.1	1,505
20–24	16.5	1,408
25–29	16.4	1,397
30–34	15.2	1,295
35–39	12.1	1,032
40–44	11.3	964
45–49	10.7	913
**Total**	100	8,514
**Place of residence**		
Urban	56.4	4,805
Rural	43.6	3,709
**Total**	100	8,514
**The highest educational attainment**		
No educ.	2.2	190
Primary	10.1	862
Secondary	77.3	6,581
Tertiary	10.3	881
**Total**	100	8,514
**Wealth index**		
Poorest	20.7	1,763
Poorer	21.9	1,865
Middle	23.0	1,956
Richer	20.4	1,733
Richest	14.1	1,197
**Total**	100	8,514
**Employment status**		
Unemployed	67.8	5,774
Employed	32.2	2,740
**Total**	100	8,514
**Provinces**		
Western Cape	7.7	656
Eastern Cape	12.2	1,041
Northern Cape	8.4	718
Free State	10.0	854
KwaZulu-Natal	16.0	1,360
North-West	10.1	863
Gauteng	10.1	863
Mpumalanga	12.4	1,054
Limpopo	13.0	1,105
**Total**	100	8,514
**Current marital status**		
Never married	60.3	5,134
Married	21.4	1,825
Living together	11.9	1,016
Widowed	2.4	202
Divorced/separated	1.1	95
Not living together	2.8	242
**Total**	100	8,514
**Race**		
Black/African participants	86.5	7,359
White participants	2.5	214
Colored participants	10.0	848
Indian/Asian participants	1.0	88
**Total**	100	8,514
**Beating wife justified**		
No	96.3	7,793
Yes	3.7	297
**Total**	100	8,090

Source: 2016 SADHS.

Table 2 presents the results showing the percentage of women who have experienced intimate-partner physical violence. The table presents chi-square statistics.

**Table 2:** Respondents who experienced intimate-partner physical violence by different socioeconomic factors.

**Variable**	**Ever experienced IPV**		**Total**	***x*^2^ *p*-value**
	**Yes (%)**	**No (%)**		
**Age groups**				
15–19	17.5	82.5	160	
20–24	13.1	86.9	627	
25–29	14.3	85.7	728	
30–34	12.2	87.8	757	0.368
35–39	12.9	87.1	619	
40–44	11.1	88.9	557	
45–49	12.1	87.9	513	
**Place of residence**				
Urban	13.3	86.7	2,301	0.302
Rural	12.2	87.8	1,660	
**Highest educational attainment**				
No educ.	12.6	87.4	972	
Primary	20.8	79.2	928	0.000
Secondary	12.8	87.2	950	
Tertiary	7.0	93.0	1,111	
**Wealth index**				
Poorest	16.7	83.3	765	
Poorer	14.1	85.9	939	
Middle	12.0	88.0	952	0.000
Richer	12.7	87.3	782	
Richest	7.1	92.9	523	
**Employment status**				
Unemployed	13.8	86.2	1,850	0.038
Employed	11.5	88.5	2,111	
**Provinces**				
Western Cape	15.8	84.2	316	
Eastern Cape	22.8	77.2	465	
Northern Cape	10.6	89.4	349	
Free State	13.9	86.1	396	
KwaZulu-Natal	9.5	90.5	535	0.000
North-West	12.5	87.5	408	
Gauteng	9.8	90.5	450	
Mpumalanga	15.3	84.7	497	
Limpopo	7.3	92.7	545	
**Current marital status**				
Never married	11.0	89.0	1,633	
Married	10.4	89.6	1,259	
Living together	19.0	81.0	738	0.000
	**Yes (%)**	**No (%)**		
Widowed	10.3	89.7	126	
Divorced/separated	22.6	77.4	62	
Not living together	22.4	77.6	143	
**Race**				
Black/African participants	13.0	87.0	3,418	
White participants	2.5	97.5	122	0.003
Colored participants	14.9	85.1	390	
Indian/Asian participants	6.9	93.1	29	
**Beating Justified**				
No	11.8	88.2	3,672	0.000
Yes	28.8	7,102	139	
**Total**	12.9	87.1	3,961	

### Chi-squares (x^2^)


$$X^{2}=\sum \frac{{\left(o-e\right)}^{2}}{e}$$

Chi square is a method used in statistics that calculates the difference between observed and expected data values (Wilson and Hilferty, 1931). It is used to determine how closely actual data fit expected data. The results were assessed using the *p*-value at a level of *p* = 0.005. The *p*-value is used to indicate whether the results are statistically significant or not, meaning is there any association between intimate-partner physical violence and socio-economic and demographic variables.

The results indicate that IPV is higher among the younger age group and gradually declines with the age of the respondents. For example, while nearly 18 and 13.1% of respondents in age group 15–19 and 20–24, respectively have experienced intimate-partner physical violence, the rate was slightly lower among the advanced age groups. Only 11.1 and 12.1% experienced this type of violence among the older age groups 40–44 and 45–49. However, these differences were not statistically significant with *p*-value of 0.368.

The results further show that only 12 and 13% of those who resided in the rural and urban spaces, respectively, had experienced violence from the intimate-partner. However, the results were not significant with *p*-value of 0.302. On the other hand, the results showed that the level of education attained by the respondents was statistically and significantly associated with intimate-partner violence (*p* < 0.0001). Highest percentages (20.8%) of study participants who experienced IPV had primary educational level followed by those with secondary and tertiary levels of education with 12.8 and 7%, respectively. The obvious conclusion then is that those with a tertiary level of education are least at risk of IPV than those with no education at all.

The results showed that in the wealth index, the poorest people were more likely to experience intimate-partner physical violence. The category of “poorest” accounts for 16.7% and those who fall under category of “poorer” accounted for 14.1% of those who experienced IPV. The proportions decline to 12.7% among those who were categorized as “Richer” and continued to decrease to 12 and 7% among those who fall under the category “Middle” and “Richest” and the differences were statistically significant (*p* < 0.0001).

Employment status has an influence on whether or not one experiences intimate-partner physical violence. In this study, most (13.8%) of those who were unemployed experienced intimate-partner physical violence as compared to those who were employed (11.5%). The results were statistically significant with *p*-value of 0.038.

Different provinces also showed interesting patterns of IPV: the results were statistically significant with a *p*-value of 0.000. Provinces which had greater number of respondents who experienced intimate-partner physical violence include Eastern Cape with 22.8%, followed by Western Cape with 15.8%, Mpumalanga with 15.3%, Free State with 13.9%, North West with 12.5%, Northern Cape with 10.6%, Gauteng with 9.8%, KwaZulu-Natal with 9.5%. The least number of respondents who experienced this form of violence were in Limpopo at 7.3%.

The variable marital status showed that the highest percentage (22.6%) of respondents that have experienced intimate-partner physical violence fall under divorced/separated category “Not living together” followed by 22.4% and “living together” with 19%. The proportion of women who were victims of intimate-partner physical violence was lower among those who were widowed (10.3%) and those who were married (10.4%). All the differences were statistically significant (*p* < 0.0001).

In examining the race of the respondents, the results reflect that IPV is statistically and significantly associated with intimate-partner violence with *p*-value of 0.003. Higher percentage of colored participants (14.9%) and Black/African participants (13%) participants were victims of intimate-partner physical violence than other races which include Indian/Asian which accounted for 6.9% and White participants 2.5% and these results were statistically significant with *p*-value of 0.003.

The perception of physical violence by an intimate partner has an influence on the actual experience of IPV. Most (28%) of the respondents who accepted that it is justified for a man to beat his wife when she argues with him were more likely to experience physical violence from the intimate partner compared to only 12% who did not agree with that statement.

Table 3 presents the odds ratios showing the likelihood of experiencing intimate-partner physical violence by different socio-economic variables. Using age group 15–19 as the reference category, the results indicated that only women in age group 20–24 were significantly associated with physical violence and were 1.8 times more likely to have experienced physical violence, and the results were marginally significant (*p* < 0.05). Under the type of residence, the results were statistically significant (*p* < 0.05). The odds ratio of experiencing intimate partner violence for those staying in the rural area was 1.28 more likely to be violated compared to women staying in the urban area.

**Table 3:** The odds ratios for experiencing intimate-partner physical violence by different socio-economic factors.

**Variable**	**B**	**Odds ratios**	**Std. error**	**Sig**.
**Age groups**				
15–19		**1.000**		
20–24	0.189	1.815^*^	0.279	0.033
25–29	0.002	1.308	0.206	0.191
30–34	0.119	1.378	0.192	0.095
35–39	0.041	1.096	0.188	0.627
40–44	0.021	1.240	0.192	0.262
45–49	0.001	1.011	0.199	0.955
**Place of residence**				
Urban^®^		**1.000**		
Rural	0.097	1.287^*^	0.126	0.046
**Highest educational attainment**				
No educ.^®^		**1.000**		
Primary	0.049	1.565	0.360	0.213
Secondary	0.358	0.334	0.237	0.000
Tertiary	0.116	0.463	0.197	0.053
**Wealth index**				
Poorest^®^		**1.000**		
Poorer	0.668	0.883^*^	0.237	0.024
Middle	0.622	0.924^*^	0.228	0.021
Richer	0.415	0.876	0.222	0.249
Richest	0.367	0.750	0.215	0.140
**Employment status**				
Unemployed^®^		**1.000**		
Employed	0.014	1.073	0.108	0.517
**Provinces**				
Western Cape^®^		**1.000**		
Eastern Cape	0.847	2.558^***^	0.266	0.000
Northern Cape	1.037	3.526^***^	0.208	0.000
Free State	0.275	1.285	0.270	0.353
KwaZulu-Natal	0.661	1.843^*^	0.239	0.011
North-West	0.087	1.344	0.231	0.200
Gauteng	0.458	1.553	0.231	0.057
Mpumalanga	0.170	1.233	0.249	0.400
Limpopo	0.568	2.014^***^	0.241	0.001
**Current marital status**				
Never married^®^		**1.000**		
Married	−0.845	0.425^***^	0.230	0.000
Living together	−0.763	0.479^***^	0.231	0.001
Widowed	−0.437	0.804	0.232	0.347
Divorced/separated	−0.689	0.422^*^	0.365	0.018
Not living together	0.145	1.374	0.383	0.278
**Race**				
Black/African participants	−0.330	**1.000**		
White participants	−0.611	1.015	0.763	0.984
Colored participants	−0.312	0.242	0.955	0.138
Indian/Asian participants		0.980	0.783	0.979
**Beating wife justified**				
No^®^	−0.894	**1.000**		0.000
Yes		3.030^***^	0.853	

Significance level: ^*^0.01–0.04, ^***^p < 0.005.

Although the results were only significant at the secondary level of education, the results of education as a variable indicated that women who had higher levels of education were 54% less likely to have experienced violence compared to those without education. Those women who had achieved secondary education were 67% less likely to have experienced physical violence from the intimate partner compared to those with no education and the results were highly significant (*p* < 0.005). Differences in other categories were found to be statistically significant.

The results show that an increase in the wealth index was negatively associated with the likelihood of experiencing physical violence (*p* < 0.05) for women who came from households categorized as are “poorer” and the “middle.” Those who were classified as “poorer” were 12% less likely to have experienced physical violence with the intimate partner compared to those who were poorest and those who were in the “middle” category were 8% less likely to experience this form of violence. All other categories, which include the richest, and richest were found not to be statistically significant (*p* > 0.05). Furthermore, while the results showed that those who were employed had an increased likelihood of experiencing IPV, these results were not statistically significant, implying that employment status has no influence on physical violence.

Regarding IPV incidents by province, the results showed that Eastern Cape, Northern Cape, KwaZulu-Natal, and Limpopo were the only provinces where the results were statistically significant compared to those who lived in Western Cape. Those staying in Eastern Cape and Northern Cape were 2.558 and 3.426, respectively more likely to have experienced IPV compared to Western Cape. In KwaZulu-Natal, participants were 1.843 more likely to have experienced intimate partner violence compared to those who live Western Cape and the results were statistically significant (*p* < 0.005) for both provinces. The Limpopo province was also found to be statistically significant (*p* < 0.05), the odds ratio for intimate physical violence in Limpopo province was 2.014 more compared to the Western Cape.

Under “Current marital status,” only three categories that include the married, living together and divorced were found to be statistically significant (*p* < 0.05). Compared to women who were never married, those who were married were 56% less likely to have experienced physical violence and those of who were “living together” were 53% less likely to have experienced intimate partner violence as compared to those who were never married. The results of the odds ratio reveal that respondents who were classified as “divorced/separated” were 58% less likely to experience this type of violence. Other categories, including widowed and not living together were found not statistically significant (*p* > 0.05).

Odds ratios (OR) for race show that the Colored participants were 76% less likely to have experienced intimate-partner physical violence, while White participants were participant 1.015 more likely to experience intimate-partner physical violence. Indian/Asian were 2% less likely to experience intimate-partner physical violence. However, all categories were found not to be statistically significant (*p* < 0.05). The justification for wife beating was found statistically significant (*p* < 0.005), showing that respondents were 3 times more likely to have experienced intimate-partner physical violence. Age group 20–24 reported (ORs = 1.815), Place of residence (Rural, ORs = 1.287), Wealth index (Poorer, ORs = 0.883, Middle, ORs = 0.924), Provinces (Eastern Cape, ORs = 2.558, Northern Cape, ORs = 3.526, KwaZulu-Natal, ORs = 1.843, Limpopo, ORs = 2.014), Current Marital Status (Married, ORs = 0.425, Living together ORs = 0.479, Divorced/separated, ORs = 0.422), and Beating wife justified (Yes, ORs = 3.030).

## Discussion

The purpose of this study was to investigate the socioeconomic factors associated with intimate-partner physical violence between intimate partners among women in South Africa. Based on the results of the study, the following major findings were observed; all other socio-economic variables which include highest educational attainment, wealth index, employment status, current marital status, race and justifying wife-beating were the main factors influencing intimate-partner physical violence. Although age and type of place of residence are the socioeconomic variables that are not major determinants of physical violence in South Africa.

As mentioned above, the study has revealed that age group was found to be statistically significant. Young age groups (20–24 years) have experienced intimate-partner physical violence but majority those at older ages (40–44 and 45–49) are less likely to be victims of intimate-partner physical violence. The National Coalition against Domestic Violence (NCADV) (2015: p. 1) stated in the literature review that young women normally have a misconception about love; they normally equate physical abuse with an expression of genuine or true love. This contrasts with the expectations derived from the findings of other scholars such as Bolarinwa et al. (2023). One contributing factor to this phenomenon may be that younger women often exhibit greater naivety regarding love, possibly perceiving physical violence as a norm in relationships. On the contrary, older women, with the benefit of more life experience, have come to realize that maintaining such naivety and expecting change may be unrealistic. Consequently, a growing number of older women are choosing to divorce or leave toxic and abusive relationships.

On contrary to these results, Bolarinwa et al. (2023) and Stephenson et al. (2022) reported that older women are prone to intimate-partner physical violence due to factors such as culture (if they are married), for instance, believing that the Bible does not allow divorce. In addition, this implies that older African women are more likely to stay in abusive relationships or marriages as a result of adhering to cultural and religious values, norms, and beliefs. This is because in African culture, older women were traditionally trained to be submissive to their husbands and exercise endurance in their relationships, even in the face of abusive dynamics. Therefore, the saying “mosadi o tshwara thipa ka fa bogaleng,” meaning a woman holds the sharp side of the knife.

There is a significant association between the type of place of residence and physical violence. Women who reside in urban areas are more likely to indicate that they have experienced physical violence than those in rural areas (Yang, 2022). On the contrary, women in rural areas seldom discuss instances of intimate-partner physical violence, primarily because societal norms often validate men's violent actions, portraying them as justified. Perpetrators in urban areas are those with a low level of employment and educational attainment, who are intimidated by the cognitive skills of their wives, participation in household's decision making of the household, occupational status and economic independence.

Based on the study, marital status was also found to be statistically significant. Women who were married and lived together strongly have experienced intimate-partner physical violence than those who were never married (Sardinha et al., 2022; Yang, 2022; Metheny, 2019; Eggers del Campo and Steinert, 2022). The decision to separate is sometimes as a result of physical aggression that she experiences, which include slapping, kicking, beating and other violent behavior (Kwagala et al., 2013: p. 1,112). This implies that men do not easily accept separation from their partners. The social expectation that women should be strong and capable of dealing with any challenge in a relationship is another factor that makes it difficult to recognize and confront intimate partner violence. The African term “*Imbokodo,”* which denotes the tenacity and strength connected to women, captures this idea (Moreira and Da Costa, 2020; Rajah and Osborn, 2022; Di Napoli et al., 2019; Ogbe et al., 2020). This cultural perspective might make it more difficult for women to confront and openly acknowledge abuse issues in their relationships. This belief also makes it difficult for men to accept separation.

The results revealed that women with tertiary education are less likely to experience intimate-partner physical violence as compared to those with no education, this is because women with tertiary education are empowered, autonomous and also more enlightened or informed about intimate-partner physical violence, its causes and consequences or effects and they also acknowledge their human rights (Wessells and Kostelny, 2022; Centers for Disease Control Prevention, 2022; Ogundipe et al., 2018; Lutgendorf, 2019; Warmling et al., 2021; Alkan et al., 2023). According to Suzane et al. (2014: p. 68) and Ranganathan et al. (2022), exposure to higher education also allows women to have a greater range of choice in partner and more ability to choose to marry or not. A man with less education than his wife may be more likely to be the perpetrator of intimate partner violence because he is intimidated by any status his wife's education may confer on her. Eggers del Campo and Steinert (2022) states that there is a need for women economic empowerment intervention which will assist those who are financially dependent to their partners. Furthermore, in the systematic review it was reported that women's economic empowerment was associated with a significant reduction in the pooled measure of emotional, sexual and physical IPV. This implies that when women are in a better financial position, they are more likely to leave abusive partners. Prevention strategies that must be implemented to reduce IPV perpetration include teaching safe and healthy relationship skills, disrupting toxic patriarchal norms, creating violent-free environments (workplace, school, home), and strengthening financial security in the household (Wessells and Kostelny, 2022; World Health Organization, 2017; Potter et al., 2021; Eggers del Campo and Steinert, 2022; Moreira and Da Costa, 2020; Rajah and Osborn, 2022; Stöckl et al., 2021; Alkan et al., 2023). The majority of participants who agreed with the statement that beating a wife is justifiable if she argues with the husband were more likely to be victims of intimate-partner physical violence. Most women support intimate-partner physical violence because they believe that a man should dominate a woman. They believe in social and cultural norms that state that a powerful man is the one who uses violence to maintain his superiority and that violence is a form of discipline and conflict resolution [UNICEF (United Nations Children's Fund), 2015: p. 8]. Traditional gender norms, which favor men over women in power dynamics (Rafael, 2017), are the basis for the idea that “REAL” men should dominate and be aggressive toward women.

Physical violence differs from one province to province. Respondents who lived in the eastern Cape, northern Cape, and Limpopo were more likely to experience intimate-partner physical violence than those residing in the western Cape. Women who live in rural Eastern Cape still carry traditional ideologies of toxic patriarchy; they are still subordinate to men and apparently may endure violent behavior in partner relationships without as much resistance to traditional socialization practices (Mesatywa, 2014: p. 14). This is more likely due to the teachings they receive as young women during *the initiation of “Intojane,”* which is the rite of passage into womanhood practiced in the Eastern Cape. During “*Intojane”* young women are taught that they should obey their man no matter what, that he should take full control of the relationship as the head of the household. This ritual might be reinforcing IPV. In a study conducted among rural, sexually active young women in the general population in the Eastern Cape Province between 2002 and 2003, there were 47% of reported physical IPV and 9% reported experiences of sexual IPV, respectively. Combined, 43% reported cases experiencing both types of IPV in the past 12 months were reported. This strongly suggests that promiscuity of women exposes their vulnerability to intimate partner violence (Zembe et al., 2015).

According to Mpani (2015: p. 18), the Northern Cape specifically, it was found that 90% of perpetrators abused alcohol or drugs. Alcohol use was recognized by communities as a key driver of intimate-partner physical violence against women. However, 66% of the participants argued that alcohol is not the only contributing factor to the violence reported. Some survivors of violence discussed experiences of violence where the perpetrator had not consumed alcohol. Other identified drivers of physical violence included social and gender inequalities, economic problems, and socialization into violent practices, and perpetrators having learnt aggression from growing up in families where violence was a common occurrence. The injuries stemming from alcohol-related violence included strangling, stabbings, and even death. According to the Gender links (2017), 77% percent of women in Limpopo province have experienced some form of physical violence during their lifetime, both within and outside intimate relationships. This is attributable to patriarchal norms, practices, and attitudes, including those that legitimate and authorize the use of violence.

The wealth index is also a determinant that influences physical violence in South Africa. Those who are not financially well off are more likely to accept that physical violence toward women is not a major issue. Most poor women are home-makers or housewives; they are the subordinates of their husbands and become submissive to their husbands, who own the financial assets to provide them with money to meet their needs and other expenses (Kim, 2016: p. 1,243). Suzane et al. (2014: p. 68) propose that unemployment in the male partner is another factor linked to the high prevalence of partner violence in South Africa. Unemployed male partners end up financially dependent on their female partners for a living. They strive to exert their superiority by enforcing physical and sexual abuse as a sign of their dominance in a patriarchal society.

## Limitations and future research directions

There are several limitations to the current study. Firstly, we used secondary data from SADHS and it did not provide us with all the answers we needed because the data were not specifically collected for our study. Secondly, we had to clean the data to match our dependent variable and other independent variables. Third, as researchers we have no control over data quality, such as missing data, incomplete surveys, recall bias, or measurement bias. The data might be lacking quality. We also need to have the appropriate skills and software (SPSS) to access, manipulate, and analyse data and analysis. Since this study focusses only on South African context, future studies might consider broadening the scope of the study by conducting a comparative study of socioeconomic factors associated with IPPV in Sub-Saharan African countries. Methodologically, qualitative perspectives and potential intervention studies must be carried out, this will contribute to a deeper understanding of perceptions, experiences, and attitudes of participants in IPPV. Research could also explore other socioeconomic determinants which were not included in this study, how they are associated with IPPV in South Africa. The other similar limitation of the paper is the assumption of linearity between the dependent variable and independent variables. This should be tested empirically and completed as a minor revision using diagnostic statistics to prove the linear relationship. Furthermore, when the relationship is not linear, the regression findings should also be amended.

## Conclusion

The burden of intimate-partner physical violence falls disproportionately on women, which is a significant concern; this is a concern. This study highlighted that there are socioeconomic factors associated with intimate-partner physical violence that include highest educational attainment, wealth index, employment status, current marital status, race and justifying wife-beating were the main factors influencing intimate-partner physical violence in South Africa. Women are at higher risk of experiencing intimate-partner physical violence in association with socioeconomic factors, therefore, there is a need to explore interventions that will help and decrease the rate of IPPV.

## Recommendations

It is recommended that more platforms are needed where young and old women will be educated about healthy relationship dynamics and signs of physical abuse; this will challenge the stereotypes about love. Women will clearly understand that love does not cause them bodily harm or injury. They should also be advised to have personal boundaries that they should not allow their partners to violate. Campaigns are needed that aim to challenge toxic gender norms and masculinity-related attitudes, which perpetuate and justifies intimate partner physical violence by intimate partners in various communities. IPPV perpetrators who witnessed the violence when growing up should be encouraged to seek professional help to address the underlying issues and break cycles of violence. The majority of women experience violence due to lack of financial independence; women should be advised to achieve financial independence; this provides them with the means to leave the abusive relationship and reclaim their freedom. Methodologically, it is recommended that future research should undertake several diagnostic tests to examine basic logistic regression assumptions. In logistic regression, basic assumptions must be met, such as the independence of errors, the absence of multicollinearity, and the lack of outliers. Additionally, recommended the use of any statistical or econometric software to investigate how socioeconomic factors contribute to IPPV and the use of regression models that can assess IPPV in the South African context.

## Data Availability

The datasets presented in this study can be found in online repositories. This data can be found here: https://www.dhsprogram.com/data/dataset/South-Africa_Standard-DHS_2016.cfm?flag=1?. The specific datasets analyzed were the “Individual Recode Dataset.” This dataset is publicly available, but requires registration for access.

## Footnotes

^1^Available at: https://www.dhsprogram.com/pubs/pdf/DHSM2/DHS6_Supervisor_Editor_Manual_30Mar2011.pdf
